# Allelopathic Substances of *Osmanthus* spp. for Developing Sustainable Agriculture

**DOI:** 10.3390/plants12020376

**Published:** 2023-01-13

**Authors:** Hisashi Kato-Noguchi, Yuri Hamada, Misuzu Kojima, Sanae Kumagai, Arihiro Iwasaki, Kiyotake Suenaga

**Affiliations:** 1 Department of Applied Biological Science, Faculty of Agriculture, Kagawa University, Miki, Kagawa 761-0795, Japan; 2 Department of Chemistry, Faculty of Science and Technology, Keio University, 3-14-1 Hiyoshi, Kohoku, Yokohama 223-8522, Japan

**Keywords:** allelochemical, 10-acetoxyligustroside, decomposition, fallen leaf, growth inhibition, *Osmanthus*, (+)-pinoresinol

## Abstract

*Osmanthus fragrans* Lour. has been cultivated for more than 2500 years because of the fragrance and color of the flowers. The flowers and roots have been used in tea, liquors, foods, and traditional Chinese medicine. The species contains more than 180 compounds including terpenoids, phenylpropanoids, polyphenols, flavonoids, and sterols. However, there has been limited information available on the allelopathic properties and allelopathic substances of *O. fragrans*. We investigated the allelopathy and allelopathic substances of *O. fragrans* and *Osmanthus heterophyllus* (G.Don) P.S. Green, as well as *Osmanthus* × *fortunei* Carrière, which is the hybrid species between *O. fragrans* and *O. heterophyllus.* The leaf extracts of *O. fragrans*, *O. heterophyllus*, and *O.* × *fortunei* suppressed the growth of cress (*Lepidium sativum* L.), alfalfa (*Medicago sativa* L.), *Lolium multiflorum* Lam., and *Vulpia myuros* (L.) C.C.Gmel with the extract concentration dependently. The extract of the hybrid species *O.* × *fortune* was the most active among the extracts. The main allelopathic substances of *O.* × *fortunei* and *O. fragrans* were isolated and identified as (+)-pinoresinol and 10-acetoxyligustroside, respectively. (+)-Pinoresinol was also found in the fallen leaves of *O.* × *fortunei*. Both compounds showed an allelopathic activity on the growth of cress and *L. multiflorum.* On the other hand, several allelopathic substances including (+)-pinoresinol may be involved in the allelopathy of *O. heterophyllus. O. fragrans*, *O. heterophyllus*, and *O.* × *fortunei* are evergreen trees. but their senescent leaves fall and cover the soil under the trees. It is possible that those allelopathic substances are liberated through the decomposition process of the leaves into their rhizosphere soil, and that they accumulate in the soil and provide a competitive advantage to the species through the inhibition of the growth of the neighboring competing plants. Therefore, the leaves of these *Osmanthus* species are allelopathic and potentially useful for weed management options in some agriculture settings to reduce commercial herbicide dependency for the developing sustainable agriculture systems.

## 1. Introduction

*Osmanthus fragrans* Lour., belonging to the Oleaceae family, is an evergreen tree that is 3–5 m high, and has been cultivated for more than 2500 years in China and some other countries because of the fragrance and color of the flowers [1,2,3]. The flowers are used in traditional tea, liquors, and foods. The essential oil is extracted for perfume. Its roots are used in traditional Chinese medicine such as in treatments for stomach ache, toothache, rheumatism, and muscle pain [4]. Pharmacological and phytochemical investigations of the chemical constituents in the plants and its essential oil suggest that the species contains more than 180 compounds, including terpenoids, phenylpropanoids, polyphenols, flavonoids, and sterols. Some of those compounds, such as ionone, flavonoids, iridoids, and polyphenols, have shown pharmacological functions through an anti-hyperglycemic activity, anti-inflammatory activity, anti-melanogenesis activity, antioxidant activity, and antitumor activity [3,5,6,7,8,9,10]. There has been limited information available on the allelopathic activity of the leaf extracts of *O. fragrans* in a publication using it as Chinese medicine [11]. However, no information is available on the compounds involved in the allelopathy of *O. fragrans*.

Allelopathy is the interaction between donor plants and receiver plants through certain secondary metabolites defined as allelochemicals, which are synthesized in some plant parts and released into the vicinity of the donner plants, including the rhizosphere soil, through the volatilization and rainfall leachates from the plants, root exudation, and decomposition process of plant residues, including fallen leaves. Allelopathy is able to provide a competitive advantage to the donner plants for resources such as nutrients, water, and light, through the inhibition of the germination and growth of the neighboring competitive plants [12,13,14,15]. The plant parts and the extracts of several plant species have shown an excellent weed control ability as mulch and soil additives due to their allelopathic characteristic [16,17,18]. Therefore, the allelopathy of plants is potentially useful for weed management options in several agriculture settings in order to reduce commercial herbicide dependency for the development of sustainable agriculture [19,20].

**Figure 1:**
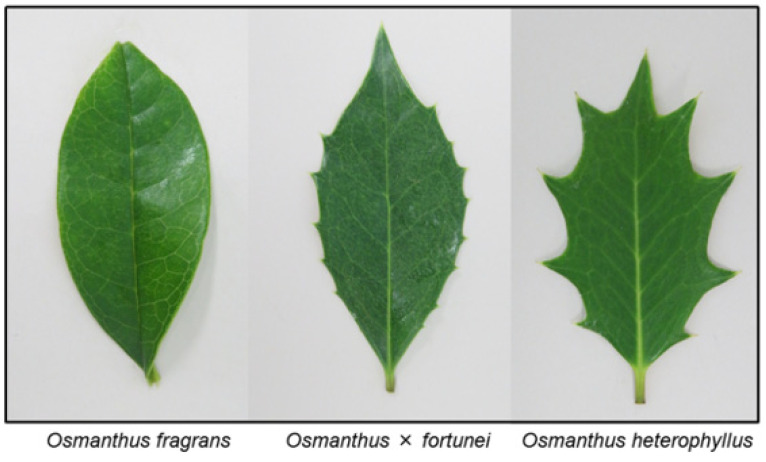
The leaves of *O. fragrans*, *O.* × *fortunei*, and *O. heterophyllus*.

**Figure 2:**
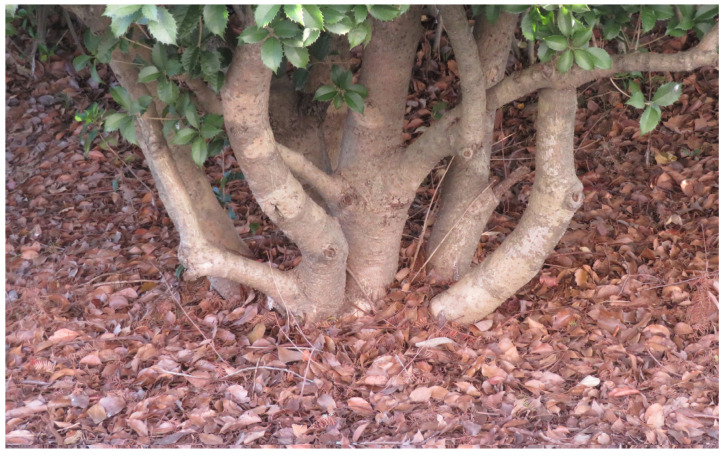
The accumulation of the fallen leaves on the soil under the tree of *O.* × *fortune*.

The *Osmanthus* genus consists of about 30 species, mostly native to Eastman Asia. *Osmanthus* × *fortunei* Carrière is a hybrid species between *O. fragrans* and *Osmanthus heterophyllus* (G.Don) P.S.Green. The leaves of *O. heterophyllus* show a serrate leaf margin and *O. fragran* leaves show no serration, and the hybrid species *O.* × *fortunei* has a combination of both of these characteristics (Figure 1). These plant species are evergreen trees but their senescent leaves fall, and the fallen leaves accumulate on the soil surfaces under the trees (Figure 2). Tree plants are able to release allelopathic substances into their rhizosphere soil over many years through the decomposition process of their fallen leaves. Those liberated compounds from the leaves may accumulate in the soil, and suppress the germination and growth of other plant species [21,22,23]. Therefore, some allelopathic substances can be released into the rhizosphere soil during the decomposition process of the fallen leaves of *O. fragrans*, *O. heterophyllus*, and *O.* × *fortune*, and act as allelopathic agents [13,14,15]. The objective of this study was the investigation of the allelopathic activity of the leaves of *O. fragrans*, *O. heterophyllus*, and *O.* × *fortune*, and the determination of the main allelopathic substances in their leaves for the development of sustainable agriculture.

## 2. Results

### 2.1. Allelopathic Activity of the Leaves of O. fragrans, O. heterophyllus and O. × fortunei 

**Figure 3:**
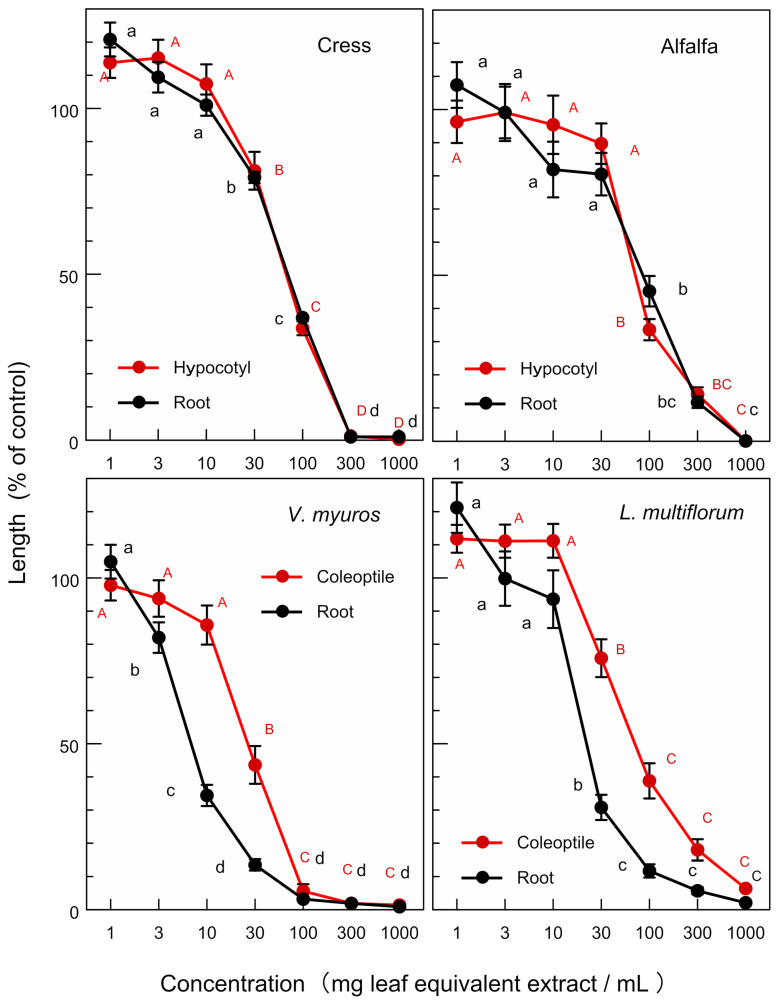
Effects of the extracts of *O. fragrans* leaves on the growth of the hypocotyls and roots of cress and alfalfa, and the growth of the coleoptiles and roots of *L. multiflorum* and *V. myuros*. Concentration (mg leaf equivalent extract/mL) indicates the concentration of the tested sample corresponding to the extracts obtained from 1, 3, 10, 30, 100, 300, and 1000 mg leaves per mL. Different letters on the symbols in the same panels indicate significant differences (Tukey’s HSD test, *p* ≤ 0.05).

**Figure 4:**
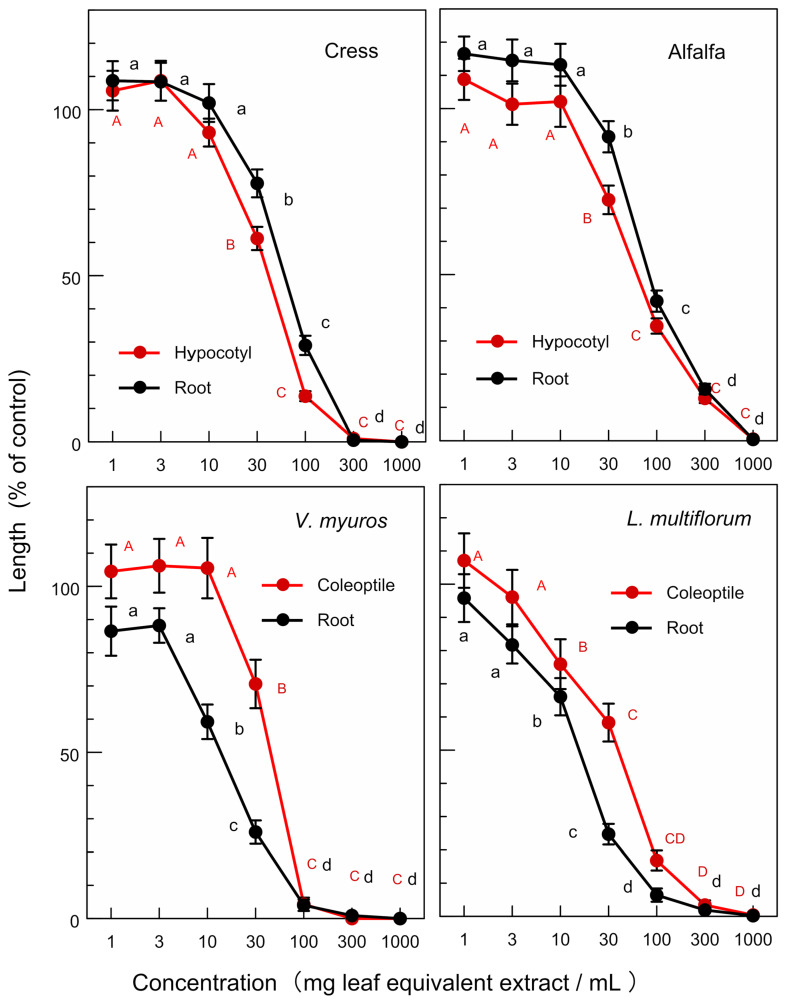
Effects of the extracts of *O. heterophyllus* leaves on the growth of the hypocotyls and roots of cress and alfalfa, and the growth of the coleoptiles and roots of *L. multiflorum* and *V. myuros*. Concentration (mg leaf equivalent extract/mL) indicates the concentration of the tested sample corresponding to the extracts obtained from 1, 3, 10, 30, 100, 300, and 1000 mg leaves per mL. Different letters on the symbols in the same panels indicate significant differences (Tukey’s HSD test, *p* ≤ 0.05).

**Figure 5:**
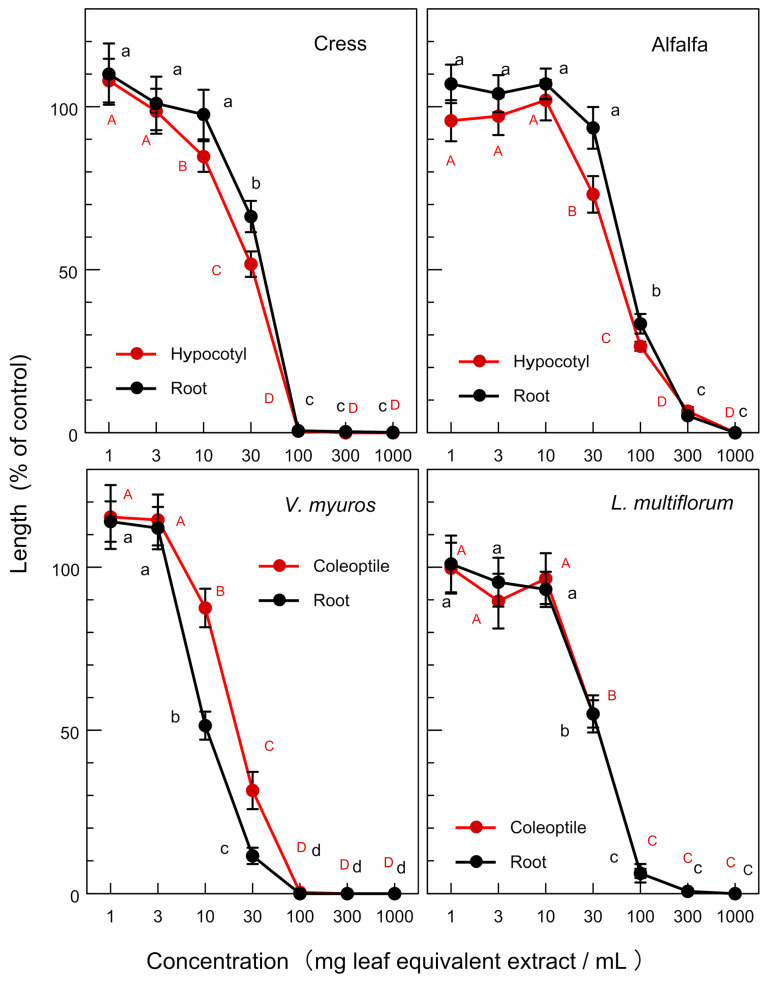
Effects of the extracts of *O.* × *fortunei* leaves on the growth of the hypocotyls and roots of cress and alfalfa, and the growth of the coleoptiles and roots of *L. multiflorum* and *V. myuros*. Concentration (mg leaf equivalent extract/mL) indicates the concentration of tested sample corresponding to the extracts obtained from 1, 3, 10, 30, 100, 300, and 1000 mg leaves per mL. Different letters on the symbols in the same panels indicate significant differences (Tukey’s HSD test, *p* ≤ 0.05).

The extracts of *O. fragrans* leaves significantly inhibited the hypocotyl and root growth of the dicotyledonous plants, namely cress and alfalfa at concentrations greater than 30–100 mg leaf equivalent extract per mL. The extract also significantly inhibited the coleoptile and root growth of the monocotyledonous weed plants, namely *V. myuros* and *L. multiflorom* at concentrations greater than 10–30 mg leaf equivalent extract per mL (Figure 3). The extracts of *O. heterophyllus* and *O.* × *fortunei* also showed an inhibitory activity on the growth of cress, alfalfa, *V. myuros*, and *L. multiflorom* (Figure 4 and Figure 5). Increasing the extract concentration of *O. fragrans*, *O. heterophyllus*, and *O.* × *fortunei* resulted in an increase in the growth suppression of the hypocotyls/coleoptiles and roots of both dicotyledonous and monocotyledonous plant species (Figure 3, Figure 4 and Figure 5).

Table 1 shows the *IC_50_* values of the extracts of *O. fragrans*, *O.* × *fortune*, and *O. heterophyllus*, which can cause 50% growth inhibition on the hypocotyls/coleoptiles and roots of the test plant species, and indicate that smaller values show a higher growth inhibitory activity than large values. All *IC_50_* values of the *O.* × *fortune* extracts, except the *IC_50_* value for *V. myuros* roots, were smaller than those of the *O. fragrans* and *O. heterophyllus* extracts.

### 2.2. Identification of Compound ***1*** and Its Activity

The extract of *O.* × *fortunei* leaves was partitioned with ethyl acetate. The activity of the obtained ethyl acetate phase was much greater than that of the aqueous phase (Table 2). Therefore, the ethyl acetate phase was separated using the chromatography of the silica gel, Sephadex LH-20, and ODS cartridge. The allelopathic activity of all of the separated fractions was determined by a cress bioassay after each chromatographic separation, as described above, and the most active fraction was carried out following chromatographic separation. Finally, active compound **1** was isolated by HPLC.

**Figure 6:**
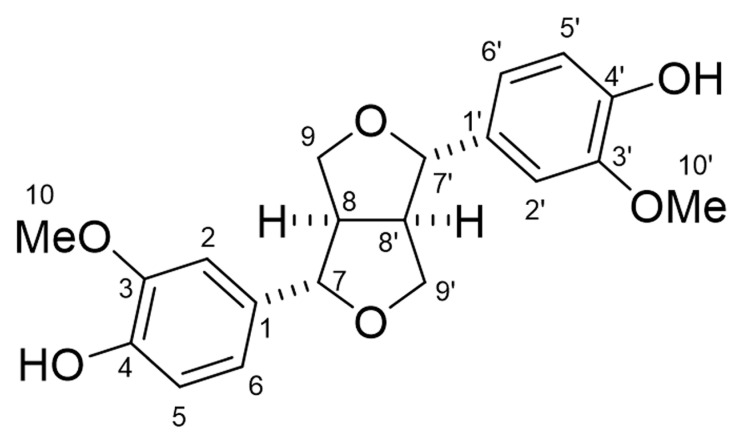
Chemical structure of (+)-pinoresinol.

**Figure 7:**
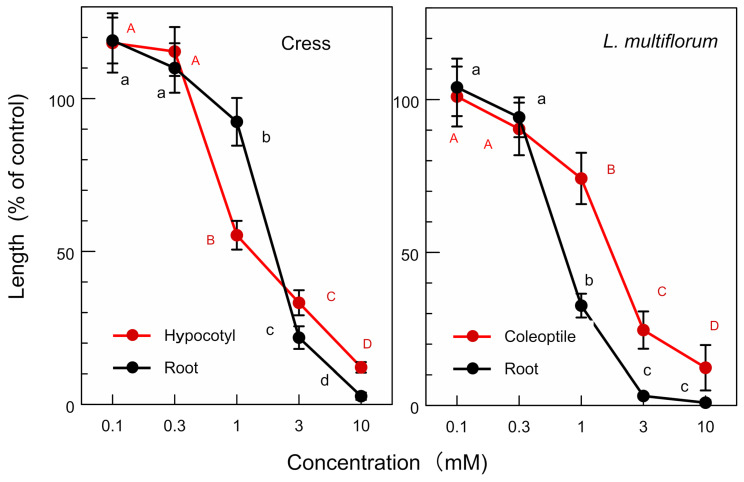
Effects of (+)-pinoresinol on the hypocotyl/coleoptile and root growth of cress and *L. multiflorum.* Means ± SE from three independent experiments with 10 seedlings for each determination are shown. Different letters on the symbols in the same panels indicate significant differences (Tukey’s HSD test, *p* ≤ 0.05).

The molecular formula of compound **1** is C_20_H_22_O_6_, as suggested by HRESIMS at *m*/*z* 359.1478 [M + H]^+^ (calcd for C_20_H_23_O_6_ 359.1495). The ^1^H NMR (400 MHz, CD_3_OD) spectrum of compound **1** showed δ_H_ 6.95 (d, *J* = 1.9 Hz, 2 H, H-2/2′), 6.81 (dd, *J* = 8.4, 1.9 Hz, 2 H, H-6/6′), 6.77 (d, *J* = 8.4 Hz, 2 H, H-5/5′), 4.71 (d, *J* = 4.5 Hz, 2 H, H-7/7′), 4.23 (dd, *J* = 9.5, 7.0 Hz, 2 H, H-9a/9a′), 3.86 (s, 6 H, H-10/10′), 3.84 (dd, *J* = 9.5, 3.9 Hz, 2 H, H-9b/9b′), and 3.14 (m, 2 H, H-8/8′). The optical rotation of the compound was [α]_D_^27^ = +84.5 (*c* = 0.215, CH_3_OH). The chemical structure of compound **1** was determined as (+)-pinoresinol (Figure 6) by the comparison of those spectra data with the published data [24]. (+)-Pinoresinol (1) significantly inhibited the growth of hypocotyls/coleoptiles and the roots of the cress and *L. multiflorum* at concentrations greater than 0.3 mM. The inhibition increased with the increasing concentration of (+)-pinoresinol (Figure 7). The *IC**_50_*** values of (+)-pinoresinol of the growth hypocotyls/coleoptiles and the roots of cress and *L. multiflorum* are shown in Table 3.

### 2.3. Concentration of (+)-Pinoresinol in the Leaves of O. × fortune, O. fragrans, and O. heterophyllus

The concentration of (+)-pinoresinol (1) in the mature leaves of *O.* × *fortune* was 4.1 mg/g and the concentration of its fallen leaves was 2.1 mg/g. However, the mature leaves *O. fragrans* and *O. heterophyllus* showed a small amount of (+)-pinoresinol (Table 4).

### 2.4. Identification of Compound **2** and Its Activity

The extract of *O. fragrans* leaves was partitioned with ethyl acetate. The activity of the obtained ethyl acetate phase was much greater than that of the aqueous phase (Table 2). The ethyl acetate phase was separated using the chromatography of the silica gel, Sephadex LH-20, and C_18_ cartridge. The allelopathic activity of all separated fractions was determined by a cress bioassay after each separation step, and the most active fraction was applied to the next separation, and an active compound **2** was finally isolated by HPLC.

**Figure 8:**
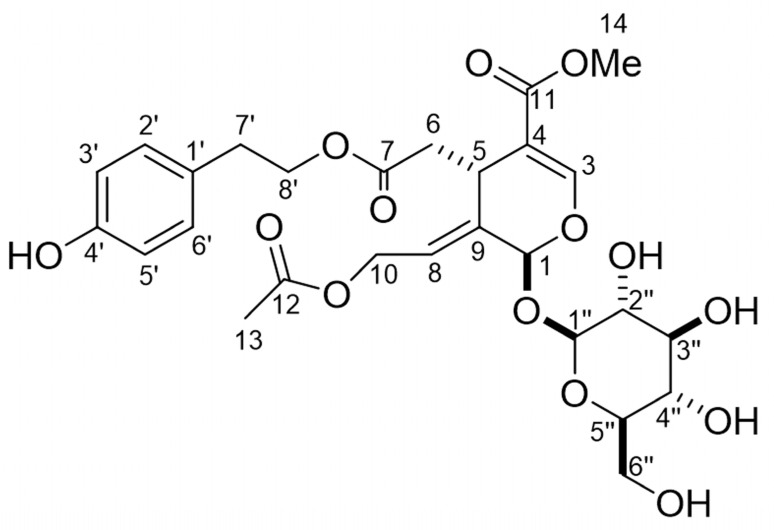
Chemical structure of 10-acetoxyligustroside.

**Figure 9:**
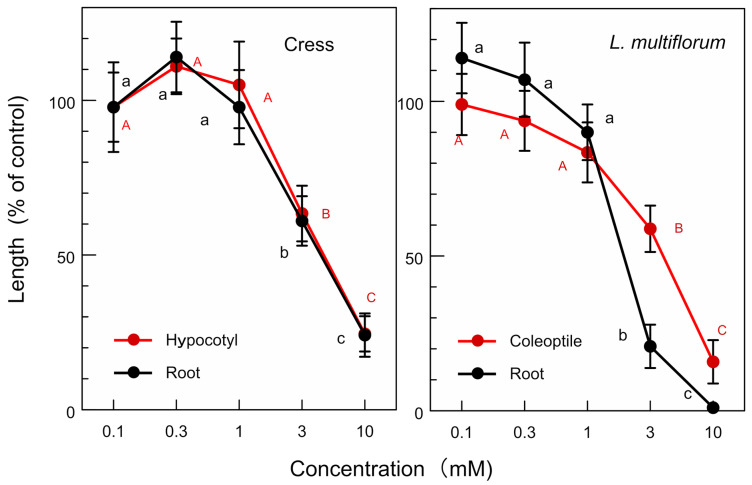
Effects of 10-acetoxyligustroside on the hypocotyl/coleoptile and root growth of cress and *L. multiflorum.* Means ± SE from three independent experiments with 10 seedlings for each determination are shown. Different letters on the symbols in the same panels indicate significant differences (Tukey’s HSD test, *p* ≤ 0.05).

The molecular formula of compound **2** is C_27_H_34_O_1_, as suggested by HRESIMS at *m*/*z* 605.1836 [M + Na]^+^ (calculated for C_27_H_34_O_14_Na, 605.1846). The ^1^H NMR (400 MHz, CD_3_OD) spectrum of the compound **2** showed δ_H_ 7.53 (s, 1 H, H3), 7.05 (d, *J* = 8.6 Hz, 2 H, H2′, H6′), 6.71 (d, *J* = 8.6 Hz, 2 H, H3′, H5′), 6.08 (brdd, *J* = 7.7, 5.9 Hz, 1 H, H8), 5.98 (brs, 1 H, H1), 4.82 (d, *J* = 7.7 Hz, 1 H, H1”), 4.76 (dd, *J* = 13.1, 7.7 Hz, 1 H, H10), 4.56 (ddd, *J* = 13.3, 5.9, 1.8 Hz, 1 H, H10), 4.24 (ddd, *J* = 10.8, 7.3, 7.3 Hz, 1 H, H8′), 4.14 (ddd, *J* = 10.8, 7.3, 7.3 Hz, 1 H, H8′), 4.00 (dd, *J* = 10.0, 4.1 Hz, 1 H, H5), 3.90 (dd, *J* = 11.8, 1.4 Hz, 1 H, H6″), 3.72 (s, 3 H, H14), 3.67 (dd, *J* = 11.8, 5.9 Hz, 1 H, H6″), 3.41 (dd, *J* = 8.6, 9.2 Hz, H3″), 3.35 (m, 1 H, H5”), 3.34 (m, 1 H, H2”) 3.33 (m, 1 H, H4″), 2.83 (t, *J* = 7.3 Hz, 1 H, H7′), 2.77 (dd, *J* = 15.4, 4.1 Hz, 1 H, H6), 2.50 (dd, *J* = 15.4, 9.5 Hz, 1 H, H6), and 2.02 (s, 3 H, H13). The ^13^C NMR (100 MHz, CD_3_OD) spectrum of the compound showed δ_C_ 172.8 (C7), 172.5 (C12), 168.3 (C11), 157.1 (C4′), 154.9 (C3), 133.9 (C9), 131.0 (C2′, C6′), 130.0 (C1′), 124.4 (C8), 116.3 (C3′, C5′), 109.1 (C4), 100.8 (C1″), 94.2 (C1), 78.5 (C3″), 77.9 (C5″), 74.7 (C2″), 71.4 (C4″), 66.9 (C8′), 62.7 (C6″), 61.8 (C10), 52.0 (C14), 41.0 (C6), 35.2 (C7′), 32.4 (C5), and 20.8 (C13). The optical rotation of the compound was [α]_D_^27^-163 (*c* 0.32, CH_3_OH). Those spectra indicate that the chemical structure of compound **2** is 10-acetoxyligustroside (Figure 8), in comparison with those reported in the literature [25]. The compound significantly inhibited the growth of the hypocotyls/coleoptiles and roots of cress and *L. multiflorum* at concentrations greater than 1 mM (Figure 9). *IC**_50_*** values of 10-acetoxyligustroside of the growth of the hypocotyls/coleoptiles and roots of cress and *L. multiflorum* are shown in Table 3.

## 3. Discussion

The leaf extracts of *O. fragrans*, *O. heterophyllus*, and *O.* × *fortunei* showed allelopathic activity against the growth of dicotyledonous plants (cress and alfalfa) and monocotyledonous weed plants (*L. multiflorum* and *V. myuros*) (Figure 3, Figure 4 and Figure 5). As allelopathic substances are synthesized and stored in some plant parts until their release into the environments [12,13,14,15], the inhibitory activity of these leaves suggest that these plant species may produce and accumulate the allelopathic substances in their leaves. All of the *IC_50_* values of *O.* × *fortunei* on the growth of the hypocotyls/coleoptiles and roots of four test plant species were the smallest compared with those of *O. fragrans* and *O. heterophyllus* except for the *IC_50_* value on the root growth of *V. myuros*. *IC_50_* values on the root growth of *V. myuros* and *O.* × *fortunei* are not significantly different (Table 1). Thus, the leaf extract of a hybrid species of *O.* × *fortunei* was the most active among the extracts of *O. fragrans*, *O. heterophyllus*, and *O.* × *fortunei*. The accumulation of the allelopathic substances in the leaves of *O.* × *fortunei* could be greater than that of the other two species. *Fallopia japonica* (Houtt.) Ronse Decraene and *Fallopia sachalinensis* (F. Schmidt) Ronse Decraene are highly competitive invasive plant species [26,27]. *F. japonica* and *F. sachalinensis* hybridize unnaturally by asexual reproduction and create a hybrid species, *Fallopia* × *bohemica* (Chrteket Chrtková) J.P. Bailey [28]. Hybridization of the both species gives genetic diversity to *F.* × *bohemica* [29,30], and the invasiveness of the hybrid species *F.* × *bohemica* is high [31,32].

The allelopathic activity is usually evaluated on the basis of the specific activity of the compound such as ppm and *IC_50_*. However, when we evaluated the contribution of the allelochemicals to the certain plant species, the concentration of the allelochemicals in the plants needs to be considered. For example, allelochemicals, which were contained in large amounts with a moderate activity, contributed more than those that contained a small amount with a very strong activity as the allelopathic agents of the plants. The concept of the total activity included both factors; the specific activity of a compound and its concentration in the plant materials, which indicates the contribution of the compounds to the allelopathy of the plants [33]. When we compare the activity of the extracts and separated fractions obtained from same weight of the plant materials, we could evaluate the total activity of the extracts and fractions, and we chose the most active extracts and fractions as described by Dr. Fujil’s group [33]. Therefore, we evaluated the activity of the separated fractions using the concept of total activity, and selected the most active separated fraction.

The most active extract of *O.* × *fortunei* leaves among the extracts of the three species was partitioned with ethyl acetate, and the ethyl acetate phase was separated as described in Section 4.4. After each chromatographic separation, the total activity of all of the separated fractions was determined by a cress bioassay, and the most active fraction was carried out next in chromatographic separation, and an active compound was isolated. The total activity of the fractions was equivalent by the plant material basis (10 and 100 mg plant materials) [33]. During the isolation process of the active compound, the cress seeds were used as the test plant because the sensitivity of the cress to the extracts was not low or high among the four test plant species (Table 1). Based on its spectrum data compared with the published data [24], the chemical structure of the isolated compound **1** was identified as (+)-pinoresinol (Figure 6). (+)-Pinoresinol may be the main contributor to the allelopathic activity of the extract of *O.* × *fortunei* leaves because the compound was isolated from the most active fraction (total activity) of every separation step.

(+)-Pinoresinol is a tetrahydrofuran lignan and is synthesized from two coniferyl alcohol monomers [34,35,36]. The compound inhibited the growth of cress and *L. multiflorum* (Figure 7), and the *IC_50_* values of the compound on the growth of the hypocotyls/coleoptiles and roots of cress and *L. multiflorum* were 0.7–2.5 mM (Table 3). The compound was also reported to show a growth inhibitory activity on the wheat coleoptiles [37], as well as an antifungal activity, antioxidative activity, and hepatoprotective activity in mammalian cells [38,39,40].

The concentration of (+)-pinoresinol in the fallen leaves of *O.* × *fortune* was less than that in its manure leaves, which indicates that degradation of the compound may occur during the senescence of the leaves or through some other factors. However, the fallen leaves of *O.* × *fortune* still contained 51% (+)-pinoresinol of its mature leaves (Table 4). *O.* × *fortune* is evergreen, but their senescent leaves cover the soil under the trees (Figure 2). The fallen leaves were gradually decomposed and decayed into the soil in the same way as other tree leaves [41,42,43]. It is possible that (+)-pinoresinol in the fallen leaves may be liberated through the decomposition process of the leaves and accumulate in their rhizosphere soil over many years. The accumulated (+)-pinoresinol may be able to suppress the germination and growth of other plant species as an allelopathic agent.

The concentration of (+)-pinoresinol in the mature leaves *O. fragrans* and *O. heterophyllus* was 0.22 mg/g and 0.12 mg/g, respectively (Table 4). It was also reported that 13 mg of (+)-pinoresinol-β-D-glucopyranoside was isolated from 2.6 kg of *O. heterophyllus* leaves [44], which is equivalent to 0.005 mg/g. The concentration of (+)-pinoresinol in the mature leaves of *O.* × *fortune* was 18.6-fold and 34.2-hfold greater than that of *O. fragrans* and *O. heterophyllus*, respectively. *O. fragrans* and *O. heterophyllus* are the parents of the hybrid species *O. × fortune*. Thus, the hybridization of both species may increase the biosynthesis of (+)-pinoresinol.

Based on the *IC_50_* values, the allelopathic activity of *O. fragrans* was 38–94% and 40–130% that of *O. × fortune* for the growth of the hypocolyls/coleoptiles and roots, respectively, of the four test plant species, and the allelopathic activity of *O. heterophyllus* was 59–72% and 60–89% that of *O. × fortune* for their growth of hypocolyls/coleoptiles and roots, respectively (Table 1). However, the concentration of (+)-pinoresinol in the mature leaves of *O. fragrans* and *O. heterophyllus* was only 5% and 3%, respectively, of that in the mature leaves of *O.* × *fortune* (Table 4). Thus, (+)-pinoresinol may not be a main allelopathic substance in the leaves of *O. fragrans* and *O. heterophyllus.*

The extract of *O. fragrans* leaves after being separated is described in Section 4.5, and an allelopathic substance was isolated and identified as 10-acetoxyligustroside (Figure 8) based on its spectrum data compared with the published data [25]. 10-Acetoxyligustroside is an iridoid glycoside and is synthesized from iridodial [45,46]. The compound inhibited the growth of cress and *L. multiflorum* (Figure 9), and the *IC_50_* values of the compound on the hypocotyls/coleoptiles and roots of the cress and *L. multiflorum* were 2.2–4.7 mM (Table 3). There is limited information about 10-acetoxyligustroside regarding the biological activity, including the allelopathic activity, in the literature. This paper is the first report showing the allelopathic activity of 10-acetoxyligustroside. In addition, 10-acetoxyligustroside may be the main contributor to the allelopathic activity of *O. fragrans* leaves because the compound was isolated from the most active fraction in every separation step.

The extract of *O. heterophyllus* leaves was also partition with ethyl acetate (Table 2), and the ethyl acetate phase was separated using the chromatography of the silica gel, Sephadex LH-20, and C_18_ cartridge for the isolation of the main allelopathic substances, similar to other leaf extracts of *O. fragrans* and *O.* × *fortunei.* However, many active fractions were identified in every separation step, and we could not isolate the main allelopathic substances from the extracts. Several lignans, neolignan glycosides, and secoiridoid glycosides were isolated from *O. heterophyllus* leaves, but their biological activities have not yet been reported [47,48,49]. There has also been no information available on the allelopathic substances in *O. heterophyllus* leaves. Considering these investigations, many allelopathic substances including (+)-pinoresinol may be involved in the allelopathy of *O. heterophyllus*. Therefore, the extracts of the leaves of these *Osmanthus* species have allelopathic potential and contain allelopathic substances, which may be species specific. However, it is necessary to determine the concentrations of these compounds in the rhizosphere soil, and to clarify the contribution of these compounds to their allelopathy.

## 4. Materials and Methods

### 4.1. Plant Material

Mature leaves of *O. fragrans* and *O. heterophyllus*, and mature and fallen leaves of *O.* × *fortunei* were obtained from the campus of Faculty of Agriculture, Kagawa University, and the nearest botanical garden. Collected leaves were weighed and kept at −25 °C. The seeds of cress (*Lepidum sativum* L.) and alfalfa (*Medicago sativa* L.) were used to determine the allelopathic activity because of their stable germination. Weed species of *Vulpia myuros* (L.) C.C. Gmel. and *Lolium multiflorum* Lam. were also used to test the allelopathic activity.

### 4.2. Extraction

Mature leaves (50 g fresh weight) of *O. fragrans*, *O. heterophyllus* and *O.* × *fortunei* were cut into small pieces and extracted separately with 80% (*v*/*v*) aqueous methanol (300 mL) for 2 days. The extracts were filtered with a filter paper (No. 2; Toyo Ltd., Tokyo, Japan), and the residue of each extract was extracted again with methanol (300 mL) for 2 days and then filtered. Two filtrates of each species were mixed and concentrated under reduced pressure at 40 °C [50]. The yield of the extracts of *O. fragrans*, *O. heterophyllus*, and *O.* × *fortunei* was 0.62, 0.57 g, and 0.69 g, respectively.

### 4.3. Determination of Allelopathic Activity

The concentrated extracts of *O. fragrans*, *O. heterophyllus*, and *O.* × *fortunei* were dissolved with methanol, and a given quantity of the methanol solution was applied to a filter paper (No. 2) in a Petri dish (2.8 cm i.d.). After the methanol solution was completely evaporated in a draft chamber, 0.6 mL of Tween 20 solution (0.05%, Nacalai, Kyoto, Japan) was added onto the filter paper. Ten seeds of alfalfa and cress and 10 germinated seeds of *L. multiflorum* and *V. myuros*, which had been germinated for 48 h in the dark at 25 °C, were separately arranged on the filter paper in the Petri dishes. After 48 h of incubation in the dark at 25 °C, the length of the roots and hypocotyls/coleoptiles of these four test plants was measured using a ruler. The percentage length of the hypocotyls/coleoptiles and roots of the test plant species treated with the leaf extracts was determined against that of each of the control seedlings. The control seedlings were treated exactly same process without the extract application [50]. The concentrations of the applied extracts in the Petri dishes were 1, 3, 10, 30, 100, 300, and 1000 mg leaf equivalent extracts per mL. The concentrations of the extract caused for the 50% growth inhibition (defined as *IC_50_*) of these test plant species were determined with GraphPad Prism 6.0 (GraphPad Software, Inc., La Jolla, CA, USA). The determination of the allelopathic activity was repeated four times using a completely randomized design with 10 plants for each determination.

### 4.4. Separation of O. × fortunei Extract

The leaves (100 g fresh weight) of *O.* × *fortunei* were extracted with 80% (*v*/*v*) aqueous methanol (600 mL) and methanol (600 mL), and were filtrated. Two filtrates were mixed and evaporated to obtain an aqueous solution, as described above. Then, the aqueous solution was made to pH 7.0 with the buffer of 0.5 M phosphate, and partitioned four times with ethyl acetate. Obtained ethyl acetate phase was concentrated to dryness, and applied to a silica gel (100 g, silica gel 60, 70–230 mesh; Nacarai, Kyoto, Japan) chromatography, which was eluted with 100 mL each of 20, 30, 40, 50, 60, 70, and 80% (*v*/*v*) ethyl acetate in *n*-hexane and ethyl acetate, and 200 mL of methanol. The allelopathic activity of those nine fractions was determined using cress seeds, as described in the Section 4.3. The activity was found in a fraction obtained by an elution of 80% ethyl acetate in *n*-hexane.

After the active fraction was evaporated, the residue was separated by a chromatography of Sephadex LH-20 (50 g, GE Healthcare, Uppsala, Sweden) and eluted with 100 mL each of 20, 30, 40, 50, 60, 70, 80, and 90% (*v*/*v*) aqueous methanol and 200 mL of methanol. An active fraction was obtained with the elution of 60% aqueous methanol, and the active residue was further separated using a reverse-phase ODS cartridge (YMC-Dispo Pack AT ODS-25; YMC Ltd., Kyoto, Japan), which was eluted with 150 mL each of 30, 40, 50, 60, 70, 80, and 90% (*v*/*v*) aqueous methanol. The active fraction was obtained by the elution with 50% aqueous methanol, and the residue was finally subjected to a reverse-phase HPLC (4.6 i.d. × 150 mm, µBondasphere 5µ C_18_-100Å, Waters Co., Ltd. (Milford, MA, USA), detection 220 nm) and eluted with 45% aqueous methanol (flow rate; 1.5 mL/min). The allelopathic activity was found in a peak fraction eluted between 125–130 min, yielding active compound **1**. The chemical structure of compound **1** was characterized by the analyses of the HRESIMS (LCT Premier XE time-of-flight TOF mass spectrometer, Waters, Tokyo) and ^1^H-NMR spectrum (400 MHz, CD_3_OD; JNM-ECX400, JEOL, Tokyo, Japan), and the optical rotation.

### 4.5. Separation of O. fragrans Extract

The leaves (500 g fresh weight) of *O. fragrans* were extracted with 80% (*v*/*v*) aqueous methanol (3 L) and methanol (3 L), and separated by silica gel chromatography, as described above. An active fraction was obtained with the elution of methanol, and the active residue was applied to the second silica gel chromatography, which was eluted with 100 mL each of ethyl acetate, and 25, 50, and 75% (*v*/*v*) acetone in ethyl acetate, acetone, and methanol. The activity was obtained by the elution of 25% acetone in ethyl acetate, and the residue was applied to a chromatography of Sephadex LH-20, and eluted with 100 mL each of 20, 30, 40, 50, 60, 70, 80, and 90% (*v*/*v*) aqueous methanol. An active fraction obtained with 30% aqueous methanol was further purified using C_18_ cartridges (YMC Ltd.), and eluted with 15 mL each of 30, 40, 50, 60, 70, 80, and 90% (*v*/*v*) aqueous methanol. The active fraction was obtained by the elution of 30% aqueous methanol. The active residue was subjected to a reverse-phase HPLC (10 i.d. × 500 mm, ODS-AQ 5µ YMC-Pack, YMC, detection 220 nm) eluted with 45% aqueous methanol (1.5 mL/min). The allelopathic activity was found in a peak fraction eluted between 104–105 min, yielding active compound **2**. The chemical structure of compound **2** was characterized by the analyses of HRESIMS and ^1^H-NMR (400 MHz, CD_3_OD), ^13^C-NMR (100 MHz, CD_3_OD) spectra and the optical rotation.

### 4.6. Allelopathic Activity of Isolated Compounds ***1*** and ***2***

Each of isolated compounds **1** and **2** was dissolved with methanol, and a given quantity of the methanol solution was added onto a filter paper in the Petri dish. After the evaporation of methanol in the Petri dishes, the filter paper was moistened with 0.6 mL Tween 20. Then, 10 seeds of cress and 10 germinated seeds of *L. multiflorum*, were separately arranged on the filter paper and incubated in the dark at 25 °C for 48 h. The allelopathic activity of the compounds and its *IC_50_* values were determined as described in Section 4.3.

### 4.7. Concentration of Compound ***1*** in the Leaves

Mature and fallen leaves (5 g) of *O.* × *fortune*, and mature leaves (5 g) of *O. fragrans* and *O. heterophyllus* were separately extracted and partitioned with ethyl acetate, as described above. The obtained residue was applied to a silica gel chromatography, and eluted with 100 mL each of 60, 70, and 80% (*v*/*v*) ethyl acetate in *n*-hexane. After evaporation of the fraction eluted with 80% ethyl acetate in *n*-hexane, the residue was applied to a reverse-phase C_18_ cartridge, and eluted with 15 mL each of 30, 40, and 50% (*v*/*v*) aqueous methanol. The fraction obtained by the elution of 40% aqueous methanol was subjected to a reverse-phase HPLC (4.6 i.d. × 250 mm, Cosmosil, Nacalai, detection 220 nm) eluted with 40% aqueous methanol (0.8 mL/min). The quantification of compound **1** was performed by the peak area on the HPLC chromatograms of samples. The recovery of compound **1** added to the leave extracts was 95 ± 11% (mean ± SE) as calculated from three replications.

### 4.8. Statistical Analysis

The data were subjected to a one-way analysis of ANOVA and subsequent post hoc analysis with Tukey’s HSD test (*p* < 0.05). *IC_50_* values were obtained using GraphPad Prism 6.0.

## 5. Conclusions

The leaf extracts of *O. fragrans, O. heterophyllus*, and *O.* × *fortunei* showed an allelopathic activity on the four test plant species, including the weed species of *Vulpia myuros* and *Lolium multiflorum.* The allelopathic substances of *O. fragrans* and *O.* × *fortunei* were isolated and identified as 10-acetoxyligustroside and (+)-pinoresinol, respectively, according to the concept of the total activity. Those compounds may be the main contributors to the allelopathy of *O. fragrans* and *O.* × *fortunei*, and provide a competitive advantage to these species through the inhibition of the growth of competing plant species in their vicinity, while several allelopathic substances, including (+)-pinoresinol, may be involved in the allelopathy of *O. heterophyllus.* The present investigation suggests that the extracts of the leaves of these *Osmanthus* species have allelopathic characteristics. Therefore, these extracts and the leaves themselves could potentially be useful for weed management options in some agriculture settings, as mulch, soil additive, and foliar spray, in order to reduce commercial herbicide dependency for the developing sustainable agriculture systems. However, it is necessary to evaluate the weed control activity of these leaves in the field condition.

## Figures and Tables

**Table 1:** *IC**_50_*** values (mg leaf equivalent extract per mL) of the extracts of the leaves of *O. fragrans*, *O.* × *fortunei*, and *O. heterophyllus* on the growth of hypocotyls/coleoptiles and the roots of bioassay plant species. *IC**_50_*** values were determined with GraphPad Prism. Different letters in the same column indicate significant differences (Tukey’s HSD test, *p* ≤ 0.05).

	*Osmanthus fragrans*		*Osmanthus* × *fortunei*		*Osmanthus heterophyllus*	
	Hypocotyl/ Coleoptile	Root	Hypocotyl/ Coleoptile	Root	Hypocotyl/ Coleoptile	Root
Cress	63.8 ± 4.2 b	63.7 ± 4.7 b	24.8 ± 1.9 c	36.5 ± 1.9 c	41.7 ± 4.1 bc	59.1 ± 4.7 b
Alfalfa	107 ± 8.1 a	153 ± 9.7 a	53.1 ± 4.2 bc	68.5 ± 7.4 b	76.5 ± 6.7 b	114 ± 9.9 a
*Lolium multiflorum*	82.6 ± 4.9 ab	30.4 ± 2.5 c	21.5 ± 1.6 c	12.4 ± 0.9 d	29.5 ± 3.1 c	13.8 ± 0.9 d
*Vulpia myuros*	25.9 ± 2.1 c	8.8 ± 0.9 d	21.4 ± 1.7 c	11.5 ± 0.9 d	36.8 ± 3.2 c	13.8 ± 1.1 d

**Table 2:** Effects of the aqueous and ethyl acetate phase obtained after the partition of the extracts of *O. fragrans*, *O. heterophyllus*, and *O.* × *fortunei* on the growth of the hypocotyl and roots of the cress (% of control). The activity was determined using the test samples obtained from 100 mg of leaf equivalent extract/mL. Different letters in the same column indicate significant differences (Tukey’s HSD test, *p* ≤ 0.05).

	*Osmanthus fragrans*		*Osmanthus* × *fortunei*		*Osmanthus heterophyllus*	
	Hypocotyl	Root	Hypocotyl	Root	Hypocotyl	Root
Aqueous phase	76.8 ± 7.5 a	101 ± 9.8 a	76.2 ± 4.3 a	62.4 ± 4.8 a	87.1 ± 4.0 a	70.9 ± 4.6 b
Ethyl acetate phase	46.8 ± 4.4 b	27.1 ± 2.7 b	22.4 ± 3.6 b	19.9 ± 3.9 b	31.9 ± 3.7 b	29.7 ± 2.9 b

**Table 3:** *IC**_50_*** values (mM) of (+)-pinoresinol and 10-acetoxyligustroside of the growth of the hypocotyls/coleoptiles and roots of the cress and *L. multiflorum. IC**_50_*** values were determined with GraphPad Prism. Different letters in the same column indicate significant differences (Tukey’s HSD test, *p* ≤ 0.05).

	(+)-Pinoresinol		10-Acetoxyligustroside	
	Hypocotyl/ Coleoptile	Root	Hypocotyl/ Coleoptile	Root
Cress	1.9 ± 0.2 a	2.5 ± 0.3 b	4.7 ± 0.3 b	4.4 ± 0.3 a
*Lolium multiflorum*	2.1 ± 0.3 a	0.7 ± 0.1 d	3.1 ± 0.3 b	2.2 ± 0.2 b

**Table 4:** The concentration of (+)-pinoresinol in the mature and fallen leaves of *O.* × *fortunei*, and in the mature leaves of *O. fragrans* and *O. heterophyllus*. Different letters indicate significant differences (Tukey’s HSD test, *p* ≤ 0.05).

*Osmanthus* × *fortunei*	(+)-Pinoresinol (mg/g)
Mature leaf	4.1 ± 0.5 a
Fallen leaf	2.1 ± 0.2 b
*Osmanthus fragrans*	0.22 ± 0.03 c
*Osmanthus heterophyllus*	0.12 ± 0.02 c

## Data Availability

No supporting data in this study.
